# Exploratory factor analysis of self-reported symptoms in a large, population-based military cohort

**DOI:** 10.1186/1471-2288-10-94

**Published:** 2010-10-15

**Authors:** Molly L Kelton, Cynthia A LeardMann, Besa Smith, Edward J Boyko, Tomoko I Hooper, Gary D Gackstetter, Paul D Bliese, Charles W Hoge, Tyler C Smith

**Affiliations:** 1 Department of Defense Center for Deployment Health Research at the Naval Health Research Center, San Diego, CA, USA; 2 Seattle Epidemiologic Research and Information Center, Veterans Affairs, Puget Sound Health Care System, Seattle, WA, USA; 3 Department of Preventive Medicine and Biometrics, Uniformed Services University of the Health Sciences, Bethesda, MD, USA; 4 Analytic Services Inc., Arlington, VA, USA; 5 Center for Psychiatry and Neuroscience, Walter Reed Army Institute of Research, Silver Spring, MD, USA

## Abstract

**Background:**

US military engagements have consistently raised concern over the array of health outcomes experienced by service members postdeployment. Exploratory factor analysis has been used in studies of 1991 Gulf War-related illnesses, and may increase understanding of symptoms and health outcomes associated with current military conflicts in Iraq and Afghanistan. The objective of this study was to use exploratory factor analysis to describe the correlations among numerous physical and psychological symptoms in terms of a smaller number of unobserved variables or factors.

**Methods:**

The Millennium Cohort Study collects extensive self-reported health data from a large, population-based military cohort, providing a unique opportunity to investigate the interrelationships of numerous physical and psychological symptoms among US military personnel. This study used data from the Millennium Cohort Study, a large, population-based military cohort. Exploratory factor analysis was used to examine the covariance structure of symptoms reported by approximately 50,000 cohort members during 2004-2006. Analyses incorporated 89 symptoms, including responses to several validated instruments embedded in the questionnaire. Techniques accommodated the categorical and sometimes incomplete nature of the survey data.

**Results:**

A 14-factor model accounted for 60 percent of the total variance in symptoms data and included factors related to several physical, psychological, and behavioral constructs. A notable finding was that many factors appeared to load in accordance with symptom co-location within the survey instrument, highlighting the difficulty in disassociating the effects of question content, location, and response format on factor structure.

**Conclusions:**

This study demonstrates the potential strengths and weaknesses of exploratory factor analysis to heighten understanding of the complex associations among symptoms. Further research is needed to investigate the relationship between factor analytic results and survey structure, as well as to assess the relationship between factor scores and key exposure variables.

## Background

Current and past US military engagements have consistently raised concern over the unique spectrum of mental and psychological symptoms experienced by deployed service members [1,2]. In particular, increased symptom reporting following the 1991 Gulf War was met with heightened effort to understand any health effects related to military service [3]. The difficulties associated with retrospective characterization and identification of etiologic factors for Gulf War-related illness highlight the importance of prospective population-based assessments of service members' health [4,5].

The Millennium Cohort Study, a 21-year longitudinal, population-based study of the health of US service members, provides a unique opportunity to investigate the interrelationships of a large number of prospectively measured physical and psychological symptoms and conditions among US military personnel [6]. While investigations of specific health exposures and outcomes among Millennium Cohort participants are ongoing [7-14], an exploratory, multivariate analysis of symptom constellations has the potential to provide new insights into service members' health. Exploratory factor analysis has been used previously in studies of 1991 Gulf War-related illnesses [15-18] and is likely to be equally valuable in understanding symptoms and health outcomes related to current US military engagements in Iraq and Afghanistan.

The main purpose of this study was to examine any underlying patterns of self-reported health symptoms provided by Millennium Cohort participants. In addition, this study aimed to determine if the questionnaire overburdens study participants and to identify a number of symptom-based factors that may be used in future studies. Exploratory factor analysis was used to describe the correlations among numerous physical and psychological symptoms in terms of a smaller number of unobserved variables or factors [19,20]. This study demonstrates the application of exploratory factor analysis to a large dataset of self-reported health symptoms in a way that addresses the categorical and sometimes incomplete nature of survey data. In addition to describing the network of relationships among health symptoms, this study may lead to the formulation of new hypotheses and a more advanced understanding of the psychometric instruments included in the questionnaires.

## Methods

### Study population and data sources

The Millennium Cohort Study Team began collecting self-reported health and exposure data in 2001. Invited participants for the first panel were taken from a stratified random sample of US military personnel serving in October 2000. Participants are surveyed every 3 years throughout a 21-year follow-up period. Using a phased enrollment strategy, the Millennium Cohort Study currently includes over 150,000 US service members. The first panel of the Millennium Cohort Study consists of 77,047 consenting participants. Fifty-five thousand and twenty (71 percent) members completed the first follow-up questionnaire between June 2004 and February 2006. More detailed descriptions of methodology for the Millennium Cohort Study are discussed elsewhere [6]. This study protocol was approved by the Institutional Review Board of the Naval Health Research Center, and the research was conducted in compliance with all applicable federal regulations governing the protection of human subjects in research (protocol NHRC.2000.007).

The population for this study consisted of Panel 1 participants who completed the baseline and first follow-up questionnaires. Participants missing values for all variables included in analyses were excluded from the study. The Millennium Cohort questionnaire asks whether participants have been diagnosed by a doctor or other medical professional with any of a number of common medical diagnoses, and also includes rare health outcomes such as kidney failure requiring dialysis, lupus, and Crohn's disease. In order to mitigate potential biases in correlation estimates, those who endorsed all 39 medical diagnosis questions were excluded from analyses, as this pattern was not medically plausible and may have indicated misinterpretation or exaggeration.

### Health and behavior metrics

The first follow-up Millennium Cohort questionnaire for Panel 1 included approximately 400 items that collected an extensive amount of information related to physical and mental health, health behaviors, deployment, occupational exposures, and other health outcomes and exposures. For this study, only responses to questions regarding physical and mental health symptoms on the first follow-up questionnaire were included in the analyses. Follow-up data were selected in order to enable future investigations of factor associations with deployment experience and other survey responses from the latter survey. A number of widely used psychometric instruments are included in the questionnaire, including the PRIME-MD Patient Health Questionnaire (PHQ) [21], the posttraumatic stress disorder (PTSD) Checklist-Civilian Version (PCL-C) [22], and the Medical Outcomes Study Short Form 36-Item Health Survey for Veterans (SF-36V) [23]. A total of 89 items that measure symptoms were factor analyzed: most items from the PHQ; all items from the PCL-C; a set of questions from the SF-36V related to bodily pain, mental health, and vitality; and 18 items derived from a study of Gulf War-related illness [3,15], hereafter referred to as the Seabee Health Questionnaire (SHQ). Question items from these embedded instruments were excluded from the analysis if they were optional, fell under a skip pattern, or did not pertain to physical or mental health symptoms. The 89 symptoms have varying dichotomous and ordinal measurement scales, with levels of possible response ranging from 2 to 6 categories. Questions pertained to time frames ranging from within 2 weeks to 3 years of survey completion.

### Statistical analysis

Exploratory factor analysis was used to determine the number and nature of factors describing the covariance structure of these data [19,20]. Because responses to questionnaire items are recorded on both dichotomous and ordinal scales, the correlation matrix for these data was estimated using polychoric correlations [24-27]. Polychoric correlations estimate the correlation between categorical variables based on the assumption that the observed categorical values are functions of latent normal random variables. Pairwise complete data were used for all correlation estimates so that all available observations for a given pair of variables were used to estimate their correlation. Analyses were also repeated on the listwise deleted population in which participants with any missing data were excluded.

Factors were extracted via the unweighted least squares factor method because it does not require that the matrix of correlation estimates be positive definite, and because, given the large number of items analyzed in this study, it is computationally more feasible than alternative estimation methods [28,29]. A number of criteria were used to determine the appropriate number of factors to retain, including the scree test [30] and Kaiser-Guttmann criterion [20], as well as requiring the root mean square residual to be less than 0.05 and requiring consecutive factors to account cumulatively for at least 75 percent of the total variation [20]. Residual correlations--specifically, requiring all residual correlations to be positive--and factor interpretability were also examined to estimate the appropriate number of factors. To enhance interpretability, orthogonal rotations were performed using the varimax procedure. Oblique rotations using the promax procedure were also considered, but orthogonally rotated solutions were the focus of this study because they greatly simplified interpretation of results, given the large number of items analyzed. Criteria for factor solution interpretability favored solutions that had at least two items with significant loadings on each factor, minimized the number of items with significant loadings on more than one factor, and for which variables loading on a common factor shared some conceptual meaning. All data manipulations were completed using SAS statistical package, version 9.2, (SAS Institute, Inc., Cary, North Carolina). Factor analyses were completed using the Mplus program, version 5.2, (Muthen & Muthen, Los Angeles, California). This research was conducted in compliance with all applicable federal regulations governing the protection of human subjects in research (protocol NHRC.2000.0007).

## Results

Of the 55,020 participants who completed the first follow-up survey, the 34 individuals who endorsed all 39 self-reported medical diagnosis questions and the 63 individuals who did not respond to any of the questions were excluded from all analyses. The demographics of the study population were nearly identical to those published elsewhere [31]: 73% male, 65% born prior to 1970, 46% with a high school education or less, 73% married, 71% white non-Hispanic, 47% active duty service members, 48% Army service members, and 11% separated from the military prior to completing the first follow-up questionnaire. Data from these participants were used to estimate a matrix of polychoric correlations between all 89 symptom variables that was used in all factor analyses. Covariance coverage values--defined as the proportion of observations used to estimate a correlation for a given pair of variables--ranged between 88 percent and 99 percent with an average value of 97 percent.

Overall, the most commonly reported symptoms were related to fatigue, trouble sleeping, and lack of energy (See additional file 1: Percentage of responses to questionnaire symptom items). Specifically, items from the SHQ that were most frequently endorsed were trouble sleeping followed by forgetfulness and severe headaches. Pain located in the back, arms, legs, or joints were the items mostly commonly reported from the PHQ somatoform items. For the depression items of the PHQ instrument, trouble sleeping or sleeping too much and feeling tired or having little energy were the most commonly endorsed items. The most frequently reported disordered eating symptom from the PHQ was inability to control the type or amount of food eaten. Being bothered by weight or personal appearance was the most highly endorsed symptom from the other PHQ items. Lack of pep and energy were the most commonly reported items of the SF-36V vitality and mental health items. While PTSD symptoms from the PCL-C and problem drinking questions from the PHQ were not highly endorsed, trouble sleeping and feeling irritable or having angry outbursts were the most frequently reported symptoms of the PCL-C items, and driving after drinking was the most commonly reported of the PHQ drinking problems.

The multiple criteria used to determine the appropriate number of retained factors suggested a wide range of models, with the number of factors ranging from 5 to 14. The scree plot and root mean square residual criteria suggested retaining 4 or 5 factors. However, the 4- and 5-factor models explained only 47 percent and 50 percent of the total variance, respectively, and did not meet the criteria for factor solution interpretability. Specifically, these models had numerous cross-loading items and variables loading on common factors lacked a shared conceptual meaning. Because of these limitations and the diversity of items included in analyses, models with higher numbers of factors were considered.

Although the Kaiser-Guttmann criterion suggested retaining 14 factors, this criterion has been criticized as somewhat arbitrary [20]; therefore, 13-, 14-, and 15-factor models were assessed. The first 15 eigenvalues for the sample correlation matrix were: 37.02, 5.95, 3.01, 2.90, 2.60, 1.82, 1.64, 1.58, 1.46, 1.37, 1.26, 1.22, 1.14, 1.03, and 0.99. Ultimately, the 14-factor model was chosen because it provided a more detailed, yet interpretable, view of the data, and because it explained 60 percent of the total variance. Due to the conflicting nature of the various model selection criteria when applied to these data, and because of the exploratory nature of this study, we chose a factor analytic solution that maximized interpretability and proportion of variance explained. This choice was in accordance with the Kaiser-Guttmann criterion, suggesting that it may be more appropriate for maximizing solution interpretability under certain circumstances. We were not able to find a factor solution that was both interpretable and explained at least 75 percent of the variance. In fact, retaining 15 factors led to a solution in which one of the factors loaded significantly with only a single variable. The 13-factor model produced more items with loadings on multiple factors than did the 14-factor model. Table 1 displays the factor loadings for the 14-factor model using a factor-loading threshold of 0.35. We varied the factor-loading threshold between 0.30 and 0.40 but selected 0.35 because it optimized factor interpretability. The root mean square residual for the final 14-factor model was 0.020 with the residual variances ranging from 0.112 to 0.738.

**Table 1:** Factor Loadings for 14-Factor Model of Symptoms Reported by Millennium Cohort Participants, 2004-2006

**Symptom description**^**a**^	**Source**^**b**^	**Factor loading**^**c**^ (× 100)	**Additional factors**^**d**^
**Factor 1 (mental health)**			
Upset when reminded of past experiences	PCL-C	84	
Physical reactions when reminded of past experiences	PCL-C	84	
Avoid thinking about past experiences	PCL-C	84	
Avoid activities that remind you of past experiences	PCL-C	84	
Repeated disturbing memories of past experiences	PCL-C	83	
Acting as if past experience is happening again	PCL-C	82	
Repeated disturbing dreams of past experiences	PCL-C	80	
Jumpy/easily startled	PCL-C	73	
Thinking/dreaming about terrible past event	PHQ (Miscellaneous)	72	
Trouble remembering parts of past experiences	PCL-C	71	
Super-alert/on guard	PCL-C	69	
Distant/cut off	PCL-C	68	
Feeling future will be cut short	PCL-C	67	
Down in the dumps	SF-36V (Vitality/Mental Health)	66	12 (38)
Emotionally numb	PCL-C	65	
Downhearted/blue	SF-36V (Vitality/Mental Health)	63	12 (37)
Loss of interest	PCL-C	61	
Difficulty concentrating	PCL-C	61	
Thoughts you would be better off dead	PHQ (Depression)	61	12 (41)
Irritable/angry	PCL-C	60	
Feel bad about yourself	PHQ (Depression)	60	12 (40)
Down, depressed	PHQ (Depression)	60	12 (45)
Nervous, anxious	PHQ (Anxiety)	60	
Nervous person	SF-36V (Vitality/Mental Health)	59	
Anxiety attack	PHQ (Panic)	58	
Moving/speaking slowly	PHQ (Depression)	58	
Trouble concentrating	PHQ (Depression)	56	
Something bad that happened recently	PHQ (Miscellaneous)	56	
No one to turn to	PHQ (Miscellaneous)	55	8 (46)
Trouble sleeping	PCL-C	52	11 (58)
Little interest/pleasure	PHQ (Depression)	52	12 (37)
Confusion	SHQ	47	13 (49)
Trouble sleeping/sleeping too much	PHQ (Depression)	45	11 (63)
Poor appetite/overeating	PHQ (Depression)	43	4 (40)
Stress at work/school	PHQ (Miscellaneous)	42	
Difficulties with spouse/partner	PHQ (Miscellaneous)	41	8 (58)
Financial problems	PHQ (Miscellaneous)	40	8 (43)
Tired/little energy	PHQ (Depression)	38	10 (45)
Stress taking care of family	PHQ (Miscellaneous)	38	8 (52)
Worn out	SF-36V (Vitality/Mental Health)	38	10 (66)
Happy person	SF-36V (Vitality/Mental Health)	-38	5 (-68)
Trouble sleeping	SHQ	37	11 (63)
Worrying about health	PHQ (Miscellaneous)	36	
Calm and peaceful	SF-36V (Vitality/Mental Health)	-36	5 (-66)
**Factor 2 (chest pain, short breath, etc.)**			
Chest pain	PHQ (Somatoform)	74	
Shortness of breath	PHQ (Somatoform)	72	
Shortness of breath	SHQ	70	3 (38)
Chest pain	SHQ	68	3 (36)
Heart pound/race	PHQ (Somatoform)	62	
Dizziness	PHQ (Somatoform)	51	
Fainting spells	PHQ (Somatoform)	49	
Unusual muscle pains	SHQ	37	3 (41), 7 (44)
**Factor 3 (flu-like symptoms)**			
Fever	SHQ	85	
Sore throat	SHQ	82	
Cough	SHQ	80	
Diarrhea	SHQ	57	9 (52)
Earlobe pain	SHQ	41	
Unusual muscle pains	SHQ	41	2 (37), 7 (44)
Night sweats	SHQ	38	
Severe headache	SHQ	38	14 (65)
Shortness of breath	SHQ	38	2 (70)
Rash/skin ulcer	SHQ	36	
Chest pain	SHQ	36	2 (68)
**Factor 4 (disordered eating)**			
Laxatives	PHQ (Disordered Eating)	75	
Made yourself vomit	PHQ (Disordered Eating)	70	
Exercised to avoid weight gain	PHQ (Disordered Eating)	70	
Can't control what/how much eat	PHQ (Disordered Eating)	68	
Fasted	PHQ (Disordered Eating)	64	
Bothered by weight/how you look	PHQ (Miscellaneous)	61	
Eat unusually large amount in 2 hours	PHQ (Disordered Eating)	53	
Poor appetite/overeating	PHQ (Depression)	40	1 (43)
**Factor 5 (vitality)**			
Lot of energy	SF-36V (Vitality/Mental Health)	-83	
Full of pep	SF-36V (Vitality/Mental Health)	-73	
Happy person	SF-36V (Vitality/Mental Health)	-68	
Calm and peaceful	SF-36V (Vitality/Mental Health)	-66	
**Factor 6 (problem drinking)**			
Drank while working	PHQ (Problem Drinking)	85	
Missed work	PHQ (Problem Drinking)	83	
Drove after drinking	PHQ (Problem Drinking)	79	
Problem getting along with others while drinking	PHQ (Problem Drinking)	70	
Drank despite doctor's warning	PHQ (Problem Drinking)	48	
**Factor 7 (aches and pains)**			
Amount of bodily pain	SF-36V (Bodily Pain)	75	
Pain in arms, legs, or joints	PHQ (Somatoform)	69	
Back pain	PHQ (Somatoform)	59	
Unusual muscle pains	SHQ	44	2 (37), 3 (41)
**Factor 8 (relationships and responsibilities)**			
Difficulties with spouse/partner	PHQ (Miscellaneous)	58	1 (41)
Stress taking care of family	PHQ (Miscellaneous)	52	1 (38)
No one to turn to	PHQ (Miscellaneous)	46	1 (55)
Little/no sexual desire/pleasure	PHQ (Miscellaneous)	44	
Financial problems	PHQ (Miscellaneous)	43	1 (40)
**Factor 9 (gastrointestinal problems)**			
Constipation, diarrhea	PHQ (Somatoform)	79	
Nausea, indigestion	PHQ (Somatoform)	62	
Stomach pain	PHQ (Somatoform)	57	
Diarrhea	SHQ	52	3 (57)
**Factor 10 (fatigue)**			
Tired	SF-36V (Vitality/Mental Health)	72	
Worn out	SF-36V (Vitality/Mental Health)	66	1 (38)
Tired/little energy	PHQ (Depression)	45	1 (38)
Sleepy all the time	SHQ	44	
Unusual fatigue	SHQ	39	
**Factor 11 (sleeping problems)**			
Trouble sleeping	SHQ	63	1 (37)
Trouble sleeping/sleeping too much	PHQ (Depression)	63	1 (45)
Trouble sleeping	PCL-C	58	1 (52)
**Factor 12 (depressed mood)**			
Down, depressed	PHQ (Depression)	45	1 (60)
Thoughts you would be better off dead	PHQ (Depression)	41	1 (61)
Feel bad about yourself	PHQ (Depression)	40	1 (60)
Down in the dumps	SF-36V (Vitality/Mental Health)	38	1 (66)
Downhearted/blue	SF-36V (Vitality/Mental Health)	37	1 (63)
Little interest/pleasure	PHQ (Depression)	37	1 (52)
**Factor 13 (cognitive problems)**			
Forgetfulness	SHQ	52	
Confusion	SHQ	49	1 (47)
**Factor 14 (headache)**			
Headaches	PHQ (Somatoform)	66	
Severe headache	SHQ	65	3 (38)

Abbreviations: PCL-C, items from the Posttraumatic Stress Disorder Checklist-Civilian Version; PHQ, items from the PRIME-MD Patient Health Questionnaire; SF-36V, items from the Short Form 36-Item Health Survey for Veterans; SHQ, items from the Seabee Health Questionnaire.

^a ^Original wording has been abbreviated and paraphrased.

^b ^Modules from the PHQ are specified as: somatoform, depression, panic, anxiety, disordered eating, miscellaneous, and problem drinking. Modules from the SF-36V are specified as: bodily pain and vitality/mental health.

^c ^Items and loadings shown for items with loading (× 100) > 35 in magnitude.

^d ^Factor number for factors with loading (× 100) > 35 in magnitude. Loading (× 100) in parentheses.

What follows is a brief description of the 14 factors from the final model (Table 1):

1. Mental health (18.5 percent of total variance). All mental health symptoms loaded on this factor. Symptoms with the highest loadings came from the PCL-C. Reporting being "upset when reminded of past experiences," having "physical reactions when reminded of past experiences," "avoiding thinking about past experiences," and "avoiding activities that remind you of past experiences" each had a factor loading of 0.84 for this factor. Having "repeated disturbing memories of past experiences," "acting as if past experience is happening again," and having "repeated disturbing dreams of past experiences" also had loadings of at least 0.80 for this factor.

2. Chest pain, short breath, etc (5.7 percent of total variance). Symptoms came from the PHQ and the SHQ. Symptoms included chest pain, shortness of breath, feeling the heart pounding or racing, dizziness, fainting spells, and unusual muscle pains. Two items about chest pain (factor loadings of 0.74 and 0.68) and two items regarding shortness of breath (factor loadings of 0.72 and 0.70) had the highest factor loadings.

3. Flu-like symptoms (5.3 percent of total variance). Symptoms came from the SHQ and included flu symptoms such as fever, sore throat, cough, and diarrhea. Reporting fever, sore throat, and cough (factor loadings of 0.85, 0.82, and 0.80 respectively) were the only factor loadings above 0.60.

4. Disordered eating (4.9 percent of total variance). All items from the PHQ used to assess disordered eating loaded on this factor. Report of using laxatives had the highest factor loading (0.75), followed by making self vomit and exercising to avoid weight gain (0.70). An item related to appetite and overeating from the PHQ depression questions also loaded on this factor with the lowest loading (0.40).

5. Vitality (3.9 percent of total variance). Symptoms were from the vitality and mental health section of the SF-36V and included having a lot of energy and feeling full of pep. All the items had high-magnitude loadings that ranged from -0.66 (feeling calm and peaceful) to -.83 (lots of energy).

6. Problem drinking (3.6 percent of total variance). All items from the PHQ designed to assess problem drinking loaded on this factor. Drank while working had the highest factor loading (0.85), while drinking despite a doctor's warning had the lowest factor loading (0.48).

7. Aches and pains (2.8 percent of total variance). Symptoms came from the SF-36V, PHQ, and SHQ, and related to bodily pain (factor loading of 0.75), pain in extremities or joints (0.69), back pain (0.59), and unusual muscle pain (0.44).

8. Relationships and responsibilities (2.8 percent of total variance). Items were from the PHQ and included being bothered by difficulties with a spouse or partner (factor loading of 0.58), stress of taking care of family members (0.52), having no one to turn to (0.46), little or no sexual desire or pleasure (0.44), and financial problems or worries (0.43). With the exception of little or no sexual desire or pleasure, all the items also loaded on factor 1.

9. Gastrointestinal problems (2.7 percent of total variance). Symptoms from the PHQ had the highest factor loadings, including constipation or diarrhea (0.79), nausea or indigestion (0.62), and stomach pain (0.57). The one symptom from the SHQ, diarrhea, had the lowest factor loading (0.52).

10. Fatigue (2.5 percent of total variance). Symptoms were from the SF-36V, the PHQ, and the SHQ. The items with the highest factor loadings, were feeling tired (0.72) and worn out (0.66). Having unusual fatigue, feeling sleepy all the time, and feeling tired or having little energy had factor loadings between 0.39 and 0.45.

11. Sleeping problems (2.2 percent of total variance). Symptoms were from the SHQ, PHQ, and PCL-C, and all were similar in nature with regard to having trouble sleeping and sleeping too much. Factor loadings were between 0.58 and 0.63.

12. Depressed mood (1.8 percent of total variance). Symptoms were from the PHQ and SF-36V, and included feeling down, depressed, or hopeless, and having suicidal or self-destructive thoughts. Factor loadings ranged between 0.37 and 0.45.

13. Cognitive problems (1.7 percent of total variance). Symptoms were from the SHQ and included forgetfulness (factor loading of 0.52) and confusion (0.49).

14. Headache (1.6 percent of total variance). Symptoms were from the PHQ and SHQ and were related to headaches with factor loadings of 0.66 and 0.65.

Analyses were repeated on the subpopulation consisting of the 35,650 individuals who had complete data for all 89 symptoms variables (results not shown). Our criteria suggested retaining the same number of factors as with the full study population, and differences between factor loadings were negligible. Additionally, obliquely rotated solutions using the promax procedure yielded a qualitatively similar factor loading matrix (results not shown).

## Conclusions

Factor analysis has been leveraged in epidemiologic research to frame broad and often complex symptom and health outcome patterns through the intercorrelations of observable symptoms and conditions. This analytical approach can be used as an exploratory tool to complement additional analyses or as a tool to understand underlying patterns in data. This study involving a large healthy military population applied exploratory factor analysis to a large dataset of self-reported symptoms, using techniques that are appropriate for binary, ordinal, and potentially incomplete data. Our exploratory analysis yielded insight into the interrelations of many self-reported physical and psychological symptoms obtained through standardized survey methods. While the factor analytic framework provided many intuitive symptom groupings, some aspects of the factor loading matrix warrant further discussion and investigation. Our finding of 14 factors that describe 60 percent of the variance of 89 variables underscores the complex set of constructs included in the Millennium Cohort questionnaire and quantifies a reasonable amount of overlap of these constructs. This assured us that the number and type of questions are appropriately assessing a spectrum of heterogeneous symptoms and conditions while affording an appreciation of the unique and shared variance of these many symptoms. These analyses also identified factors that may be used in more focused epidemiologic studies of specific exposure-outcome relationships.

The most significant factor in explaining the total variation in symptoms data was the "mental health" factor, which accounted for nearly 19 percent of the total variance. It is noteworthy that nearly all variables related to mental health outcomes loaded on a single factor, with several items from the PCL-C loading most significantly. This phenomenon persisted across multiple models with differing numbers of retained factors so that, from the perspective of factor analysis, the outcomes of depression, anxiety disorder, panic disorder, and PTSD do not represent distinct constructs in this general military population sample.

The fact that almost all the mental health symptoms loaded on a common factor can be interpreted from both a clinical and methodological framework. A clinical interpretation of this factor highlights the high degree of co-morbidity among mental health disorders. From a methodological perspective, however, these results also suggest inherent problems with the application of factor analysis across several survey instruments specific for individual clinical conditions. It is difficult to rule out the possibility that the structure of the survey influenced the factor analytic results, since many of the mental health questions are located adjacent to one another on the survey. It is possible that the factor structure reflects both underlying clinical phenomena as well as survey structure, since the factors appear to be organized according to both content and question sequence. Although exploratory factor analysis did not distinguish between many of the mental disorders assessed by the PHQ and the PCL-C, it did identify disordered eating symptoms as constituting a distinct construct (factor 4). This makes sense, given that the PHQ includes a specific disordered eating module and there is less overlap in these symptoms with symptoms of depression and anxiety disorders. "Depressed mood" (factor 12), involving items from two different instruments, also showed some degree of specificity as a distinct construct.

A number of symptoms suggestive of cardiovascular disease characterize factor 2, including chest pain and shortness of breath from the PHQ and SHQ. Overall the frequency of several of these symptoms was low, with the proportion of subjects bothered "a lot" by these symptoms being less than 2 percent as assessed by the PHQ. Most of the symptoms (chest pain, shortness of breath, fainting/dizziness, and heart pounding) loading on Factor 2 are well-recognized somatic symptoms that accompany an anxiety disorder [32]. The likelihood of cardiovascular disease is low for several reasons, including the younger age distribution of this population, the fact that all had to pass the military induction physical in order to serve, and because all were fit enough to be on active duty in the military during initial sampling in October 2000. Muscle pain is not usually associated with anxiety disorder and this analysis suggests that it could be a manifestation of it or another condition associated with anxiety such as fibromyalgia [33].

Factor 3 comprises "persistent or recurring" symptoms reported on the SHQ commonly associated with viral and bacterial infections of the respiratory and gastrointestinal tract. Earlobe pain possibly was interpreted by respondents to mean earache, which is also frequently associated with respiratory infections due to otitis media or auditory tube dysfunction. The majority of these symptoms were reported infrequently (less than 10 percent of respondents). Recurring viral infections would still be very compatible with these symptom loadings as upper respiratory infections, such as the common cold, typically occur several times in any given year [34].

Factor 5, "vitality," loaded with symptoms from the SF-36V related to energy and mood. This factor also suggests both a clinical and methodological interpretation, since all four variables that characterize this factor occur in the same section of the survey instrument. However, factor 10, "fatigue," also related to energy level and loaded with items from several different sections of the survey. The fact that two factors, accounting cumulatively for 6.4 percent of the variance, related to energy level, vitality, and fatigue suggests that further research may be needed to understand the importance of these symptoms in military populations. Factor 11, "sleeping problems," also highlights this issue. Furthermore, cross-loadings between factors 10 and 11 could indicate underlying clinical sleep disorders in this population. Previous research has found that Cohort members report an adjusted average sleep time of 6.5 hours per night [35], which is slightly lower than most recommendations for optimum sleep duration. Over a prolonged period of time such sleep deficits could be manifested in fatigue and lack of energy, among other symptoms, and result in lasting effects on performance.

All five variables from the PHQ modules pertaining to alcohol abuse loaded significantly on factor 6, "problem drinking." The highest loading variables related to drinking and work, driving, or social interactions. The last variable, "drank despite doctor's warnings," had a more modest loading, which may reflect that problem drinking affects many domains before it is addressed by physicians.

Four variables from different instruments loaded on Factor 7. We named Factor 7 "aches and pains," because each of the four variables was designed to assess general myalgia. The four variables from the survey instrument included questions about experiencing bodily pain, pain associated with arms and legs, back pain, or unusual muscle pain. Muscle pain is a common symptom, especially in an active, athletic military population. However, general muscle pain can accompany many other illnesses, such as infectious diseases, autoimmune disorders, fibromyalgia, as well as other medical conditions, including comorbid psychiatric disorders. It is interesting to note that variables related to arthralgia or other joint-related pain did not load on Factor 7, nor did headache pain.

Factor 8 included variables linked to relationship and responsibility issues from one module of the PHQ. The five variables that loaded on Factor 8 include having difficulties with a spouse or partner, experiencing stress from taking care of family members, feeling as if there were no one to turn to, having little or no sexual desire/pleasure, and experiencing financial problems. With the exception of financial problems, each of the variables is related to human interaction and communication. However, underlying psychological issues and life stressors could also contribute to how these variables group.

The last two factors had the fewest number of significantly loading variables. Forgetfulness and confusion had significant loadings on Factor 13, "cognitive problems," and were notably grouped together in the survey (SHQ) following distinctly physical symptoms. Confusion also loaded equally on Factor 1, "mental health."

Factor 14 includes headache and severe headache variables and may reflect, at least in part, that headache can be a singularly incapacitating symptom. Severe headache items also loaded on Factor 3, "flu-like symptoms," but only weakly, and may be related to grouping with other variables in the survey instrument that loaded on that factor.

There are several significant limitations to this study. While invited participants were a random weighted sample of the US military, the study population may not be representative of the entire US military population. However, foundational investigations of potential biases in the Millennium Cohort have found the cohort to be representative, with participants who report data reliably [6,9,31,36-41]. Although the Millennium Cohort Study is a longitudinal study, this exploratory analysis is based on a cross-sectional examination of the symptoms reported during a single follow-up period so that temporal associations cannot be established. Furthermore, this analysis does not address potentially significant associations between exposure and demographic variables with factor structure. Future investigations will examine the relationship of deployment histories and other exposure variables with covariance structure and factor scores associated with the current model. All symptoms and diagnoses included in this study are self-reported, and, therefore, are imperfect surrogates for clinical diagnoses [31,36-38].

Despite these limitations, this study has a number of important strengths. To our knowledge, this is the first study to perform an exploratory factor analysis of this size in a large population-based cohort of US military personnel. The large sample size allowed for the inclusion of rare symptoms while minimizing the risk of biased correlation estimates [27]. Factor analysis is an inherently subjective method, as different accepted criteria for model building may lead to disparate results. However, in order to examine the sensitivity of results to our methodological choices, analyses were repeated using multiple rotation procedures and applying several criteria for determining the number of factors. Although analyses were conducted using pairwise complete data, results from analyses repeated on a subset of the study population with complete data indicate that missing data did not influence our results.

An important finding of this study was that the majority of the factors appeared to load strongly based on how symptoms were grouped according to location on the survey. Item location, content, and response format are highly correlated with one another on the Millennium Cohort questionnaire, and this may have explained the factor loading that was observed. Thus, a major limitation of our study was that it was not able to differentiate the relative contributions of item content, location, and response format to factor loadings. This was particularly notable for mental health items, in which there was minimal ability to distinguish between individual mental disorders. This finding suggests that factor analysis may have major limitations when applied to surveys that contain several discrete validated instruments that use different response patterns and group questions according to diagnosis or co-locate questions pertaining to each domain as part of a larger survey. Further research is needed to determine how best to apply factor analysis across multiple illness domains. For example, surveys that randomly allocate symptom items across the survey and standardize response patterns could be compared with traditional surveys that include discrete disease-specific modules.

Understanding the full spectrum of symptoms and illness in a population includes investigating the interrelation of many comorbidities. Exploratory factor analysis is one way to study many symptoms and health outcomes comprehensively and to develop insight into the interrelations of symptom and outcome complexes that should be considered for future study. This study demonstrates a robust exploratory factor analysis including binary, ordinal, and some incomplete data to describe 14 factors accounting for 60 percent of the variance of 89 variables. This study also highlighted a complex set of constructs included in the survey instrument, a reasonable amount of overlap of the constructs, and assured us that the number and type of questions were appropriately assessing a spectrum of heterogeneous symptoms. Results further suggest that additional research is needed to investigate the relationship between factor analytic results and survey structure. Future research may also include the longitudinal examination of stable and evolving comorbidity structures and their relationship with self-reported exposures and health behaviors, as well as demographic and military-specific characteristics.

## List of Abbreviations

PCL-C: Posttraumatic Stress Disorder Checklist-Civilian Version; PHQ: Patient Health Questionnaire; PTSD: posttraumatic stress disorder; SF-36V: Short Form 36-Item Health Survey for Veterans; SHQ: Seabee Health Questionnaire.

## Competing interests

The authors declare that they have no competing interests.

## Authors' contributions

All authors contributed to study concept and design. MK conducted the literature review, performed the analyses, prepared major portions of the draft manuscript, and edited the final version of the manuscript. CL acquired the data and drafted sections of the draft manuscript. BS supervised the collection of data and prepared parts of the conclusions. EB, TH, and GG drafted major parts of the conclusions. PB provided key assistance to many of the technical aspects of the analysis. CH initially suggested the study, contributed to the interpretation of results, and prepared parts of the conclusions. TS supervised all aspects of the study and prepared parts of the conclusions. All authors interpreted the data, revised the article critically for important intellectual content, and approved the final version.

## Pre-publication history

The pre-publication history for this paper can be accessed here:

http://www.biomedcentral.com/1471-2288/10/94/prepub

## Supplementary Material

Additional file 1 **Percentage of Responses to Questionnaire Symptom Items by Question Source Among Millennium Cohort Participants, 2004-2006**. Indicates the percentage of responses for each of the 89 mental health symptoms. Click here for file
