# Supplementary material for: Exploratory factor analysis of self-reported symptoms in a large, population-based military cohort
Source: BMC Med Res Methodol. 2010 Oct 15;10:94. doi: 10.1186/1471-2288-10-94 (PMC2967557; doi:10.1186/1471-2288-10-94)
Supplement: Additional file 1 — Percentage of Responses to Questionnaire Symptom Items by Question Source Among Millennium Cohort Participants, 2004-2006. Indicates the percentage of responses for each of the 89 mental health symptoms. [file 1471-2288-10-94-S1.DOC]

**Percentage of Responses to Questionnaire Symptom Items by Question Source Among Millennium Cohort Participants, 2004-2006**

|  | **Response level** | | | | | | |
| --- | --- | --- | --- | --- | --- | --- | --- |
| **Symptom description**^a^ | *n*^b^ | % | % | % | % | % | % |
| **SHQ**^c^ |  | **No** | **Yes** |  |  |  |  |
| Severe headache | 54,069 | 83.1 | 16.9 |  |  |  |  |
| Diarrhea | 54,002 | 90.0 | 10.0 |  |  |  |  |
| Rash/skin ulcer | 53,872 | 91.9 | 8.1 |  |  |  |  |
| Sore throat | 53,853 | 90.9 | 9.2 |  |  |  |  |
| Bladder infections | 53,786 | 98.3 | 1.7 |  |  |  |  |
| Cough | 53,893 | 89.2 | 10.8 |  |  |  |  |
| Fever | 53,657 | 96.3 | 3.7 |  |  |  |  |
| Hair loss | 53,743 | 97.3 | 2.7 |  |  |  |  |
| Earlobe pain | 53,726 | 98.7 | 1.3 |  |  |  |  |
| Sleepy all the time | 53,705 | 86.9 | 13.1 |  |  |  |  |
| Night sweats | 52,572 | 90.3 | 9.7 |  |  |  |  |
| Chest pain | 52,587 | 93.3 | 6.7 |  |  |  |  |
| Unusual muscle pains | 52,598 | 89.3 | 10.7 |  |  |  |  |
| Shortness of breath | 52,566 | 91.4 | 8.6 |  |  |  |  |
| Trouble sleeping | 52,648 | 76.7 | 23.3 |  |  |  |  |
| Unusual fatigue | 52,542 | 84.4 | 15.6 |  |  |  |  |
| Forgetfulness | 52,538 | 82.0 | 18.0 |  |  |  |  |
| Confusion | 52,426 | 95.0 | 5.1 |  |  |  |  |

| **PHQ Somatoform**^d^ |  | **Not bothered** | **Bothered a little** | **Bothered a lot** |  |  |  |
| --- | --- | --- | --- | --- | --- | --- | --- |
| Stomach pain | 54,043 | 84.9 | 12.6 | 2.5 |  |  |  |
| Back pain | 54,175 | 52.2 | 34.5 | 13.3 |  |  |  |
| Pain in arms, legs, or joints | 54,119 | 51.1 | 33.3 | 15.6 |  |  |  |
| Problems during sexual intercourse | 53,981 | 92.6 | 5.6 | 1.8 |  |  |  |
| Headaches | 54,139 | 68.7 | 25.2 | 6.1 |  |  |  |
| Chest pain | 54,105 | 92.8 | 6.4 | 0.8 |  |  |  |
| Dizziness | 54,086 | 91.1 | 8.0 | 0.9 |  |  |  |
| Fainting spells | 54,068 | 98.9 | 1.0 | 0.1 |  |  |  |
| Heart pound/race | 54,100 | 88.5 | 10.2 | 1.4 |  |  |  |
| Shortness of breath | 54,008 | 89.4 | 9.1 | 1.5 |  |  |  |
| Constipation, diarrhea | 53,996 | 78.2 | 17.6 | 4.3 |  |  |  |
| Nausea, indigestion | 54,019 | 76.3 | 18.8 | 5.0 |  |  |  |
|  |  |  |  |  |  |  |  |
| **PHQ Depression**^e^ |  | **Not at all** | **Several days** | **More than half the days** | **Nearly every day** |  |  |
| Little interest/pleasure | 54,212 | 80.3 | 15.0 | 3.0 | 1.7 |  |  |
| Down, depressed | 54,126 | 81.1 | 14.6 | 2.9 | 1.5 |  |  |
| Trouble sleeping/sleeping too much | 54,022 | 68.4 | 20.8 | 6.4 | 4.5 |  |  |
| Tired/little energy | 54,034 | 61.8 | 28.4 | 6.3 | 3.6 |  |  |
| Poor appetite/overeating | 53,907 | 78.8 | 14.4 | 4.4 | 2.5 |  |  |
| Feel bad about yourself | 54,091 | 84.8 | 10.8 | 2.7 | 1.8 |  |  |
| Trouble concentrating | 54,058 | 85.5 | 10.6 | 2.5 | 1.5 |  |  |
| Moving/speaking slowly | 54,040 | 94.0 | 4.2 | 1.1 | 0.6 |  |  |
| Thoughts you would be better off dead | 54,100 | 97.2 | 2.0 | 0.4 | 0.3 |  |  |
|  |  |  |  |  |  |  |  |
| **PHQ Panic**^f^ |  | **No** | **Yes** |  |  |  |  |
| Anxiety attack | 50,408 | 94.3 | 5.7 |  |  |  |  |
|  |  |  |  |  |  |  |  |
| **PHQ Anxiety**^g^ |  | **Not at all** | **Several days** | **More than half the days** |  |  |  |
| Nervous, anxious | 50,317 | 67.9 | 26.8 | 5.3 |  |  |  |
|  |  |  |  |  |  |  |  |
| **PHQ Disordered Eating**^h^ |  | **No** | **Yes** |  |  |  |  |
| Can’t control what/how much eat | 54,405 | 88.3 | 11.7 |  |  |  |  |
| Eat unusually large amount in 2 hours | 54,363 | 92.3 | 7.7 |  |  |  |  |
| Made yourself vomit | 54,247 | 99.4 | 0.6 |  |  |  |  |
| Laxatives | 54,268 | 99.4 | 0.6 |  |  |  |  |
| Fasted | 54,227 | 96.4 | 3.6 |  |  |  |  |
| Exercised to avoid weight gain | 54,229 | 97.2 | 2.8 |  |  |  |  |
|  |  |  |  |  |  |  |  |
| **PHQ Miscellaneous**^i^ |  | **Not bothered** | **Bothered a little** | **Bothered a lot** |  |  |  |
| Worrying about health | 54,372 | 61.4 | 33.3 | 5.3 |  |  |  |
| Bothered by weight/how you look | 54,383 | 44.7 | 43.2 | 12.2 |  |  |  |
| Little/no sexual desire/pleasure | 54,185 | 82.6 | 13.1 | 4.4 |  |  |  |
| Difficulties with spouse/partner | 54,232 | 72.6 | 20.8 | 6.6 |  |  |  |
| Stress taking care of family | 54,297 | 71.5 | 23.5 | 5.0 |  |  |  |
| Stress at work/school | 54,317 | 59.8 | 31.9 | 8.2 |  |  |  |
| Financial problems | 54,306 | 61.9 | 30.2 | 7.9 |  |  |  |
| No one to turn to | 54,256 | 82.0 | 13.5 | 4.5 |  |  |  |
| Something bad that happened recently | 54,149 | 83.6 | 11.5 | 4.9 |  |  |  |
| Thinking/dreaming about terrible past event | 53,958 | 90.5 | 7.2 | 2.3 |  |  |  |
|  |  |  |  |  |  |  |  |
| **PCL-C**^j^ |  | **Not at all** | **A little bit** | **Moderately** | **Quite a bit** | **Extremely** |  |
| Repeated disturbing memories of past experiences | 53,930 | 78.8 | 14.5 | 3.8 | 2.1 | 0.8 |  |
| Repeated disturbing dreams of past experiences | 53,901 | 86.2 | 9.2 | 2.6 | 1.4 | 0.6 |  |
| Acting as if past experience is happening again | 53,794 | 86.2 | 9.1 | 2.7 | 1.5 | 0.5 |  |
| Upset when reminded of past experiences | 53,847 | 81.5 | 12.3 | 3.5 | 1.9 | 0.9 |  |
| Trouble remembering parts of past experiences | 53,844 | 90.5 | 5.8 | 2.1 | 1.1 | 0.5 |  |
| Loss of interest | 53,903 | 75.9 | 15.8 | 4.5 | 2.6 | 1.1 |  |
| Distant/cut off | 53,820 | 73.2 | 17.3 | 4.9 | 3.1 | 1.5 |  |
| Emotionally numb | 53,869 | 81.9 | 10.8 | 3.8 | 2.2 | 1.3 |  |
| Feeling future will be cut short | 53,811 | 85.9 | 8.6 | 2.7 | 1.7 | 1.1 |  |
| Trouble sleeping | 53,883 | 63.3 | 20.8 | 7.4 | 5.5 | 3.0 |  |
| Irritable/angry | 53,850 | 65.2 | 23.5 | 6.5 | 3.3 | 1.6 |  |
| Difficulty concentrating | 53,851 | 71.3 | 19.9 | 5.1 | 2.7 | 1.2 |  |
| Super-alert/on guard | 53,797 | 83.5 | 9.7 | 3.6 | 2.0 | 1.2 |  |
| Jumpy/easily startled | 53,757 | 85.0 | 9.7 | 3.0 | 1.5 | 0.8 |  |
| Physical reactions when reminded of past experiences | 53,707 | 89.3 | 7.0 | 2.0 | 1.1 | 0.6 |  |
| Avoid thinking about past experiences | 53,708 | 86.0 | 8.7 | 2.8 | 1.6 | 0.9 |  |
| Avoid activities that remind you of past experiences | 53,442 | 88.8 | 6.8 | 2.4 | 1.2 | 0.9 |  |
|  |  |  |  |  |  |  |  |
| **SF-36V Bodily Pain**^k^ |  | **None** | **Very mild** | **Mild** | **Moderate** | **Severe** | **Very severe** |
| Amount of bodily pain | 54,279 | 23.4 | 35.7 | 21.3 | 15.2 | 3.6 | 0.7 |
|  |  |  |  |  |  |  |  |
| **SF-36V Vitality/Mental Health**^l^ |  | **None of the time** | **A little of the time** | **Some of the time** | **A good bit of the time** | **Most of the time** | **All of the time** |
| Full of pep | 54,293 | 9.5 | 16.2 | 27.4 | 22.4 | 22.8 | 1.7 |
| Nervous person | 54,325 | 70.4 | 20.3 | 6.0 | 2.0 | 0.9 | 0.4 |
| Down in the dumps | 54,156 | 79.5 | 13.2 | 4.4 | 1.7 | 0.9 | 0.3 |
| Calm and peaceful | 54,287 | 5.9 | 12.8 | 22.4 | 24.7 | 30.9 | 3.3 |
| Lot of energy | 54,056 | 7.1 | 15.9 | 26.8 | 23.7 | 24.3 | 2.3 |
| Downhearted/blue | 54,273 | 58.7 | 28.7 | 8.3 | 2.7 | 1.2 | 0.4 |
| Worn out | 54,202 | 20.6 | 40.1 | 24.0 | 9.5 | 4.3 | 1.5 |
| Happy person | 54,279 | 2.8 | 9.0 | 16.2 | 21.0 | 43.2 | 7.9 |
| Tired | 54,281 | 9.0 | 40.3 | 30.5 | 11.7 | 6.2 | 2.4 |
|  |  |  |  |  |  |  |  |
| **PHQ Problem Drinking**^m^ |  | **No** | **Yes** |  |  |  |  |
| Drank despite doctor’s warning | 54,367 | 99.1 | 0.9 |  |  |  |  |
| Drank while working | 54,388 | 97.2 | 2.8 |  |  |  |  |
| Missed work | 54,327 | 98.8 | 1.2 |  |  |  |  |
| Problem getting along with others while drinking | 54,342 | 98.6 | 1.4 |  |  |  |  |
| Drove after drinking | 54,248 | 96.1 | 3.9 |  |  |  |  |

Abbreviations: PCL-C, items from the Posttraumatic Stress Disorder Checklist-Civilian Version; PHQ, items from the PRIME-MD Patient Health Questionnaire; SF-36V, items from the Short Form 36-Item Health Survey for Veterans; SHQ, items from the Seabee Health Questionnaire.

^a^ Original wording has been abbreviated and paraphrased. Items are listed in the order in which they appear on the questionnaire.

^b^ Number of participants who responded to question.

^c^ Question states, “In the last 3 years, have you had persistent or recurring problems with any of the following?”

^d^ Question states, “During the last 4 weeks, how much have you been bothered by any of the following problems?”

^e^ Question states, “Over the last 2 weeks, how often have you been bothered by any of the following problems?”

^f^ Question states, “In the last 4 weeks, have you had an anxiety attack – suddenly feeling fear or panic?”

^g^ Question states, “Over the last 4 weeks, how often have you been bothered by any of the following problems?”

^h^ Questions either begin with, “Do you often…?” or “In the last 3 months, have you done…?”

^i^ Question states, “In the last 4 weeks, how much have you been bothered by any of the following problems?”

^j^ Question states, “In the past month have you experienced…?”

^k^ Question states, “During the past 4 weeks, how much bodily pain have you had?”

^l^ Question states, “During the past 4 weeks, how much of the time:”

^m^ Question states, “In the last 12 months, have any of the following happened to you more than once?”
